# Gut Microbiota‐Linked Benefits of Low‐Intensity Pulsed Ultrasound Rejuvenate the Ageing Muscle

**DOI:** 10.1002/jcsm.70291

**Published:** 2026-04-21

**Authors:** Jia‐Hua Jhuang, Kuo‐Cheng Lan, Ting‐Yu Chang, Ding‐Cheng Chan, Shing‐Hwa Liu

**Affiliations:** ^1^ Institute of Toxicology, College of Medicine National Taiwan University Taipei Taiwan; ^2^ Department of Emergency Medicine Tri‐Service General Hospital, National Defense Medical University Taipei Taiwan; ^3^ Department of Geriatrics and Gerontology, College of Medicine and Hospital National Taiwan University Taipei Taiwan; ^4^ Department of Medical Research China Medical University Hospital, China Medical University Taichung Taiwan; ^5^ Department of Pediatrics, College of Medicine and Hospital National Taiwan University Taipei Taiwan

**Keywords:** gut microbiota, LIPUS, naturally ageing, skeletal muscle

## Abstract

**Background:**

Ageing is an inevitable biological process that contributes to increased prevalence of age‐associated diseases, including sarcopenia, defined by progressive loss of muscle mass, functional decline and a heightened risk of injury. Developing effective interventions remains a critical clinical priority. This study employed a natural ageing mouse model to investigate whether noninvasive low‐intensity pulsed ultrasound (LIPUS), a therapeutic ultrasound, delivered to the abdomen, could alleviate age‐related muscle deterioration and whether its effects were linked to gut microbiota modulation.

**Methods:**

C57BL/6 mice were maintained until 92 weeks of age, after which abdominal LIPUS stimulation was administered for 8 weeks. At 100 weeks, both forelimb and hind limb grip strength were assessed prior to euthanasia. Faecal samples from the distal colon were collected for microbiota profiling, and gastrocnemius muscles were harvested for downstream analyses.

**Results:**

Naturally aged mice exhibited sarcopenia‐like characteristics, including impaired muscle performance, reduced myofiber diameter and decreased muscle weight (*n* = 6, *p* < 0.01, *p* < 0.001). Age‐related renal impairment promoted the accumulation of advanced glycation end products (AGEs) in skeletal muscle, triggering pro‐inflammatory signalling cascades characterized by elevated COX‐2, phosphorylated NF‐κB, NLRP3, IL‐1β and Caspase‐1 (*n* = 5–6, *p* < 0.01). LIPUS treatment significantly improved muscle strength (forelimb and hind limb grip strength, *n* = 6, *p* < 0.001, p < 0.01) and muscle mass (*n* = 6, *p* < 0.01), while suppressing inflammatory mediators (*n* = 5–6, *p* < 0.05). Gut microbiota analysis showed that LIPUS increased microbial diversity (*n* = 5–6, *p* < 0.05) and altered taxonomic composition, enriching anti‐inflammatory taxa such as Lactobacillus, Bifidobacterium, Faecalibaculum and Coriobacteriaceae_UCG_002 (*n* = 6, p < 0.05). Correlation analysis indicated that these LIPUS‐enriched taxa were positively associated with enhanced muscle performance. These data suggest that LIPUS mitigates sarcopenia in naturally aged mice by restoring muscle integrity and attenuating inflammation, possibly via gut microbiota regulation.

**Conclusions:**

This study shows that natural ageing in mice induces sarcopenia‐like features with inflammatory activation and gut microbiota alterations. Abdominal LIPUS treatment alleviated muscle loss, reduced inflammation and promoted beneficial microbes, rejuvenating the ageing muscle. These findings highlight LIPUS as a safe, noninvasive and potentially translatable strategy for sarcopenia, warranting further investigation of its microbiota–muscle interactions.

## 1 Introduction

The steadily rising global life expectancy, together with ongoing advances in medical care, has led to a marked expansion of the older adult population. Current data indicate that in 2019, approximately 703 million people worldwide were aged 65 years or older, and this figure is expected to nearly double, reaching 1.5 billion by 2050 [1]. Ageing is closely linked to a gradual deterioration of multiple physiological systems and organ functions [2]. Importantly, the rapid acceleration of population ageing in recent decades has been paralleled by a notable surge in age‐related disorders. Accordingly, the search for effective strategies to prevent or attenuate ageing‐associated diseases has become increasingly urgent. Among these conditions, sarcopenia stands out as one of the most widespread and clinically relevant syndromes. Sarcopenia is a progressive disorder characterized by ongoing loss of skeletal muscle mass and function, ultimately resulting in adverse outcomes including functional impairment, reduced quality of life and elevated mortality risk [3].

Current management of sarcopenia typically relies on a combination of exercise and nutritional support [4], with resistance training remaining the primary approach due to its documented ability to improve muscle mass and strength [5]. However, drug‐based therapies for sarcopenia are still scarce [4], underscoring the need for innovative treatment options. Low‐intensity pulsed ultrasound (LIPUS), a noninvasive technique that transmits gentle pulsed acoustic energy with minimal heat generation, has demonstrated beneficial effects in animal models and clinical trials, including attenuation of muscle wasting in chronic kidney disease [6], stimulation of soft‐tissue repair [7] and reduction of intramuscular fat via PIEZO1 upregulation [8]. Unlike earlier investigations that mainly used injury or disease animal models, the present study employs a natural ageing mouse model to more accurately simulate human ageing. The therapeutic potential of LIPUS in this context remains insufficiently characterized and merits further study.

In parallel, research on the gut microbiota has advanced considerably, particularly regarding the interactions along the gut–muscle axis [3]. An increasing body of evidence indicates that shifts in gut microbial communities can influence skeletal muscle mass and function through various mechanisms, including protein metabolism, chronic low‐grade inflammation, metabolic resistance (diminished responsiveness to metabolic regulation) and mitochondrial dysfunction [9]. Ageing is known to profoundly reshape gut microbial structure and disturb host immune and inflammatory balance. These microbial changes may regulate immune activity by altering the equilibrium between pro‐ and anti‐inflammatory pathways [10]. A well‐balanced gut microbiota contributes to immune homeostasis, suppresses chronic inflammation and strengthens gut barrier integrity by modulating mucosal immune responses [9]. Based on this rationale, our study applied abdominal LIPUS in a natural ageing mouse model to determine whether it can reshape gut microbiota composition and thereby rejuvenate the ageing muscle and mitigate ageing‐related sarcopenic features.

## 2 Methods

### 2.1 Animal Study

Male C57BL/6 mice aged 92 weeks (old) and 4 weeks (young) were obtained from the National Laboratory Animal Center and housed under controlled environmental conditions (22°C ± 2°C; 12 h light/dark cycle) with ad libitum access to food and water. Ninety‐two‐week‐old mice were randomly assigned to either an untreated old group or an old + LIPUS treatment group (*n* = 6 per group). Four‐week‐old mice served as the young control group (*n* = 6). LIPUS was administered to the abdominal region once daily for 20 min at an intensity of 0.3 W/cm^2^ for eight consecutive weeks. Mice were sacrificed at 100 weeks (old groups) and 12 weeks (young group) of age.

All experimental procedures for mice were approved by the Institutional Animal Care and Use Committee of the National Taiwan University College of Medicine (IACUC Approval No. 20201067), and the animal study was conducted according to the guidelines for the care and use of laboratory animals.

### 2.2 Grip Strength Test

Forelimb and hind limb grip strength were measured using a Digitech grip strength metre (DTG‐50 N, Osaka, Japan). For forelimb assessment, mice grasped a horizontal metal bar with their forepaws; hind limb strength (kicking force) was recorded by positioning the hind limbs against a resistance platform. Each mouse underwent five consecutive measurements, and the mean value was calculated to ensure accuracy and reliability. Grip strength was expressed in newton (N).

### 2.3 Histological Analysis

Gastrocnemius muscles were excised from the hind limbs and fixed in 4% paraformaldehyde. Samples were paraffin‐embedded, sectioned at 5 μm and stained with haematoxylin and eosin (H&E) or processed for immunohistochemistry (IHC) or immunofluorescence (IF). Cross‐sectional area (CSA) and myofiber number were evaluated under high‐power fields using a Nikon Eclipse TS100 microscope equipped with a Canon EOS R50 digital camera. Measurements were taken from five randomly selected fields per section, and CSA was determined on 150–250 muscle fibres per mouse using ImageJ software (version 1.53, NIH).

For IHC, sections were dewaxed, rehydrated, blocked and incubated overnight at 4°C with primary antibodies against advanced glycation end products (AGE), the AGE receptor (RAGE), Caspase‐1 (P10), MuRF1, Atrogin‐1, Bax and Bcl‐xL. Haematoxylin was used for counterstaining. Semiquantitative analysis of IHC staining was performed with ImageJ.

For IF, sections were deparaffinized, rehydrated and subjected to heat‐induced antigen retrieval using EDTA buffer (pH = 9). After blocking, multiplex immunofluorescence staining was performed using a Four‐Colour TSA Detection Kit for Rabbit/Mouse Primary Antibodies (catalogue no. PK10032) according to the manufacturer's instructions. Sections were incubated with primary antibodies (Table S1) for 1 h at RT, followed by horseradish peroxidase (HRP)‐conjugated secondary antibodies and tyramide signal amplification. Between each staining cycle, antigen stripping was performed to allow sequential labelling. Nuclei were counterstained with DAPI. Multiplex immunofluorescence‐stained sections were imaged using the TissueFAXS imaging system (TissueGnostics, Vienna, Austria). Quantitative image analysis was performed using the StrataQuest Analysis System (TissueGnostics). Nuclei were identified based on DAPI staining, and individual cells were segmented accordingly. Quantification was performed using a single‐cell–based analysis. Fluorescent signals associated with DAPI‐positive nuclei were defined as positive cells. DAPI staining served as the reference for total cell number. The percentage of Ly6G^+^, F4/80^+^ and CD3^+^ cells was calculated relative to the total number of DAPI^+^ nuclei within the analysed tissue area.

For frozen sections, muscle samples were fixed in 4% paraformaldehyde and cryoprotected in 30% sucrose to prevent ice crystal formation. Tissues were embedded in optimal cutting temperature (OCT) compound and cryosectioned at 6–7 μm for fluorescent immunostaining. Sections were washed twice with PBS containing 2% BSA, fixed in 10% formalin for 10 min and rewashed. Permeabilization was performed with 0.1% Triton X‐100 in PBS for 10 min, followed by blocking in PBS containing 2% BSA for at least 1 h at room temperature. After applying a hydrophobic barrier around the tissue, sections were incubated overnight at 4°C with primary antibodies against MyHC I, MyHC IIa, and Lamin B1 diluted in PBS with 2% BSA. After 24 h, sections were incubated with fluorescent secondary antibodies (CF 568 Goat anti‐Rabbit and CF 488A Goat anti‐Mouse; Biotium) for 1 h at room temperature, washed twice with PBS and mounted with Fluoromount‐G for imaging. Antibody details are provided in Table S1.

### 2.4 Immunoblotting

Gastrocnemius samples were homogenized and lysed in RIPA buffer. After centrifugation at 13000 rpm, supernatants were collected for protein quantification. Equal amounts of protein (20 μg) were separated by 12% SDS‐PAGE and electrotransferred onto PVDF membranes. Membranes were blocked with 5% nonfat milk at room temperature for 1 h and incubated overnight at 4°C with primary antibodies against MyHC I, MyHC IIa, p21, p53, phosphorylated (p)‐NFκB, NFκB, NLRP3, IL‐1β, COX‐2, Atrogin‐1, MuRF1, Bax, Bcl‐xL, Bcl‐2, β‐actin and GAPDH (Table S1). After three washes with TBST, membranes were incubated with HRP‐conjugated secondary antibodies for 1 h at room temperature. Bands were visualized by enhanced chemiluminescence and quantified by densitometry using ImageJ software.

### 2.5 Real‐Time Quantitative Polymerase Chain Reaction Analysis

Total RNA at a concentration of 1 μg was isolated from gastrocnemius muscle using TRIzol reagent (Invitrogen, Carlsbad, CA, USA). The extracted RNA was reverse transcribed into first‐strand complementary DNA using 200 units of MMLV reverse transcriptase (Invitrogen) in a total reaction volume of 20 μL. Quantitative real‐time polymerase chain reaction analysis was performed using a SYBR Green detection system with self‐designed primers. Primer sequences for Ly6g6c, Adgre1, Cd3e and Rpl13a used in this study are shown in Table S4. Polymerase chain reaction amplification was carried out using a two‐step cycling protocol consisting of denaturation at 95°C for 15 s, followed by annealing and extension at 60°C for 1 min, for a total of 40 cycles on StepOne and StepOnePlus real‐time PCR systems (Applied Biosystems). Relative messenger RNA expression levels were calculated using the 2^‐ΔΔCt^ method with Rpl13a serving as the internal control.

### 2.6 Faecal Microbiota Analysis

Mouse faecal samples were collected and sent to TrustGene for genomic DNA extraction and sequencing. The 16S/18S/ITS rDNA amplicon sequencing workflow included quality control, amplification of variable regions with specific primers, library construction and high‐throughput sequencing on Illumina or MGI platforms. Paired‐end sequencing (up to 600 bp reads) enhanced taxonomic resolution. Rigorous quality control and library normalization ensured data accuracy. Resulting sequences were analysed to profile microbial community composition and abundance.

Raw sequencing data were processed through quality filtering, de‐splicing and chimera removal to obtain valid sequences, which were then clustered or denoised to generate OTUs (Operational Taxonomic Units) or ASVs (Amplicon Sequence Variants) followed by taxonomic annotation. Based on OTU/ASV data, α‐diversity and community richness/evenness were calculated. Community structure was further analysed using taxonomic profiling, NMDS (nonmetric multidimensional scaling) analysis and PCoA (Principal Coordinates Analysis) visualization. Subsequent analyses included group‐wise community comparisons, associations with environmental factors and identification of key drivers of microbial composition.

### 2.7 Statistical Analysis

Differences between two groups were assessed using unpaired two‐tailed *t*‐tests, while multiple group comparisons were performed with one‐way ANOVA followed by Tukey's post hoc test. Data are presented as mean ± SD. Statistical analyses were conducted in GraphPad Prism (version 9.0), and *p* < 0.05 was considered statistically significant.

## 3 Results

### 3.1 Effect of LIPUS on Relative Organ Weights, Muscle Function and Biochemical Parameters

**FIGURE 1:**
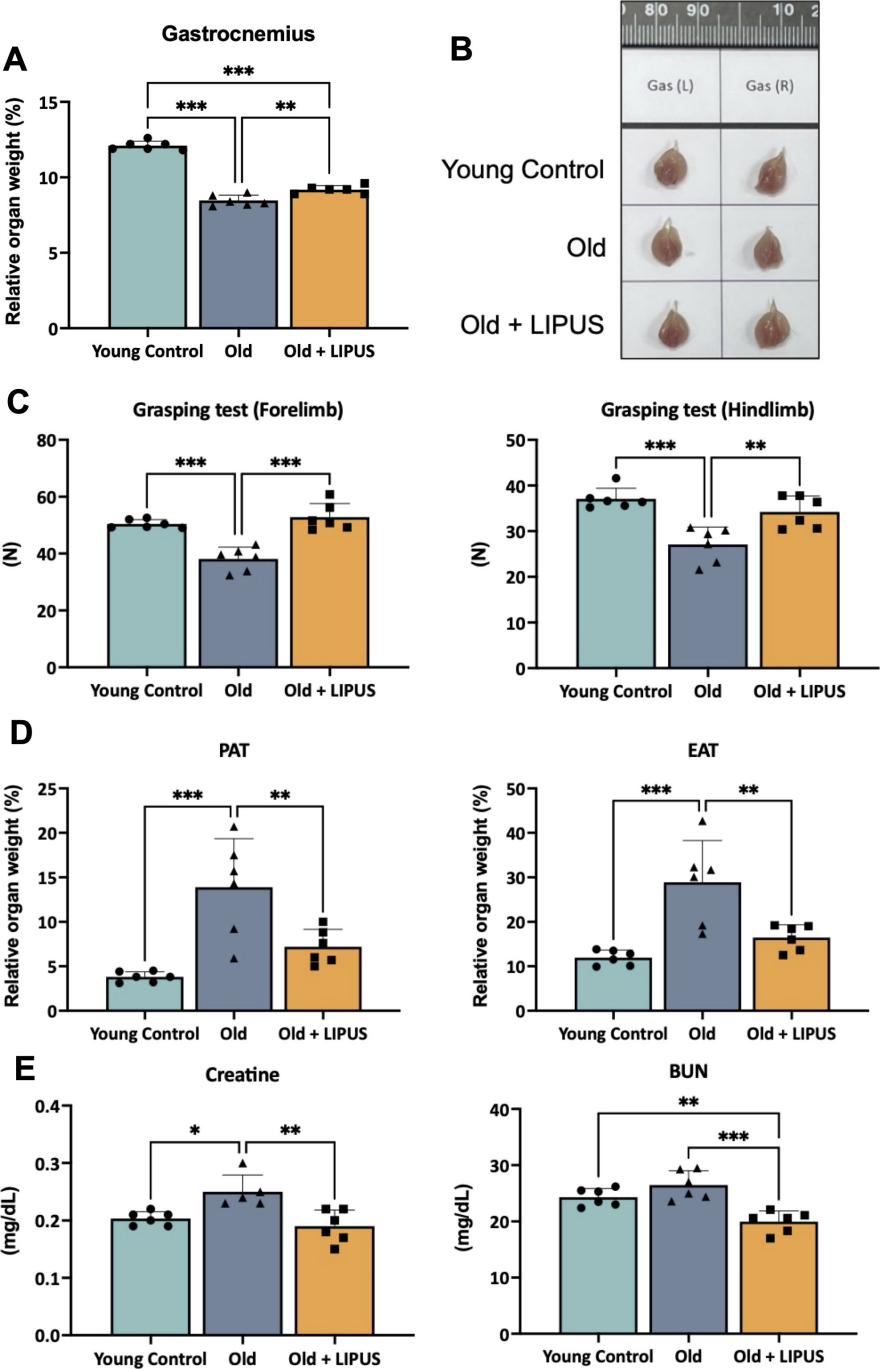
LIPUS improved organ weight, muscle function and serum biochemical parameters in aged mice. C57BL/6 mice at 92 weeks of age were treated with LIPUS for 8 weeks before sacrifice. (A) Relative weight of the gastrocnemius. (B) Gross morphology of the gastrocnemius. (C) Grasping test to assess muscle function, including forelimb and hindlimb strength. (D) Relative organ weight of perirenal adipose tissue (PAT) and epididymal adipose tissue (EAT). (E) Serum biochemical parameters related to kidney function. *, *p* < 0.05; **, *p* < 0.01; ***, *p* < 0.001.

A natural ageing mouse model was employed to examine the impact of abdominal LIPUS on age‐related skeletal muscle loss and functional decline. The body weight significantly increased in aged mice with or without LIPUS treatment (Figure S1A). The kidney weight of aged mice significantly increased, but the relative kidney weight was not changed in aged mice with or without LIPUS treatment (Figure S1B). The relative gastrocnemius muscle weight was significantly lower in aged mice compared with young controls, but this reduction was partially, but significantly, reversed by LIPUS treatment (Figure 1A). Morphological observation of gastrocnemius muscle showed a pronounced decrease in muscle volume in the aged group compared with both the young and LIPUS‐treated groups (Figure 1B). Grip strength testing revealed markedly reduced forelimb and hind limb strength in aged mice, whereas LIPUS administration restored strength toward youthful levels (Figure 1C). Perirenal and epididymal fat depots were significantly heavier in aged mice but decreased after LIPUS exposure, implying a possible effect of LIPUS on lipid metabolism (Figure 1D). Moreover, serum analysis showed elevated creatinine in the aged group versus the young control group; LIPUS significantly lowered both creatinine and BUN levels compared with the aged group, indicating a possible mitigation of LIPUS on age‐related kidney dysfunction (Figure 1E).

### 3.2 LIPUS Increased Fascicle Cross‐Sectional Area and Altered Muscle Fibre Type

**FIGURE 2:**
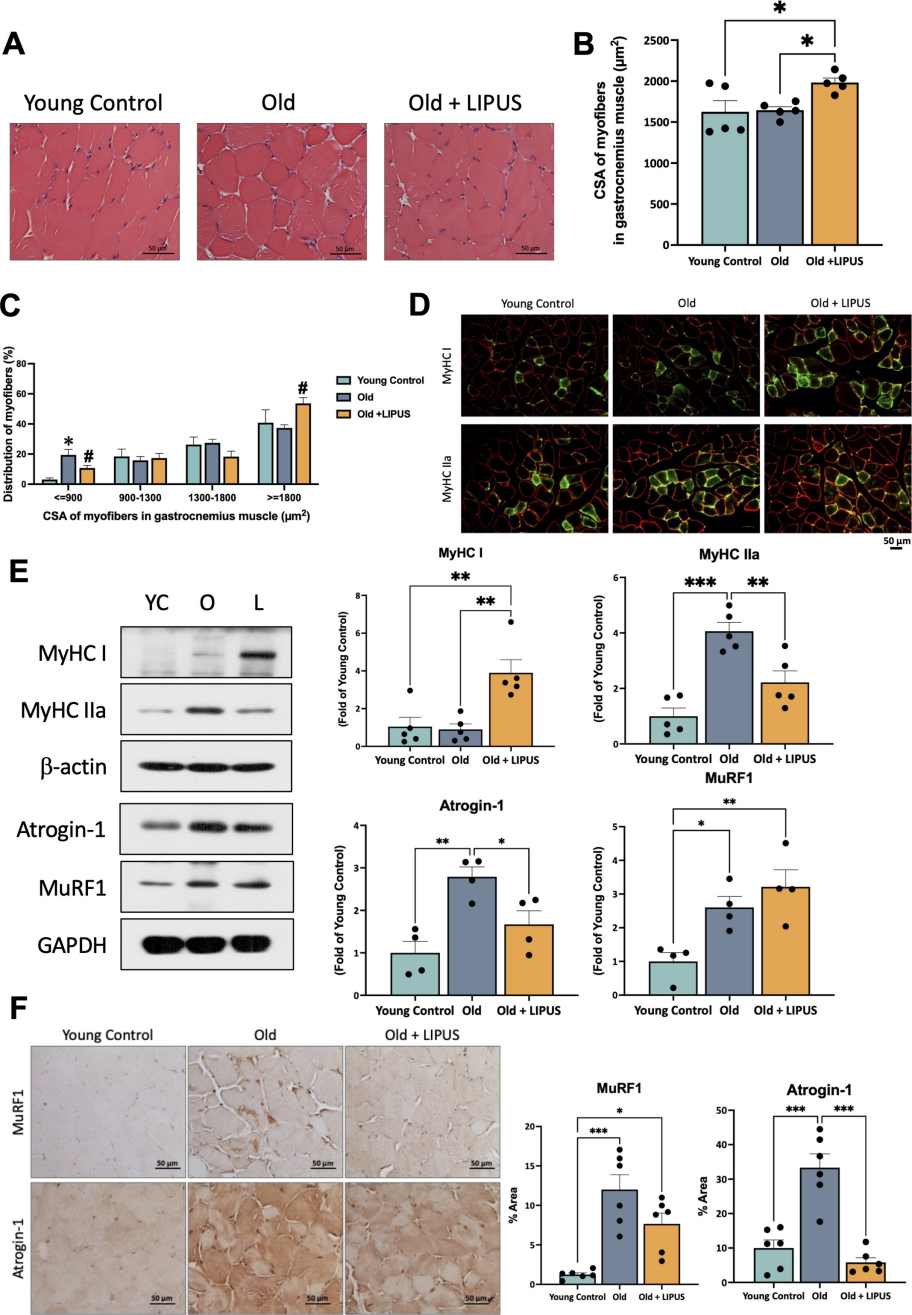
Effects of LIPUS on myofiber cross‐sectional area, fibre size distribution and changes in muscle type and atrophy markers. Gastrocnemius were harvested and processed for histological and immunofluorescence (IF) analyses. Paraffin‐embedded sections were stained with haematoxylin and eosin (H&E) and IHC, while frozen sections were subjected to IF staining. (A) Representative cross‐sectional image of Gas (40× objective lens, scale bar = 50 μm). (B) Quantification of myofiber cross‐sectional area and (C) distribution. (D) IF staining showing the distribution of MyHC type IIa and type I fibres (20× objective lens, scale bar = 50 μm). (E) Representative Western blot images and corresponding quantitative analysis of MyHC Type IIa and Type I fibres, as well as Atrogin‐1 and MuRF1 protein expression in the gastrocnemius. (F) Representative IHC images and corresponding quantification of Atrogin‐1 and MuRF1 (40× objective lens, scale bar = 50 μm). *, *p* < 0.05; **, *p* < 0.01; ***, *p* < 0.001.

Because LIPUS alleviated age‐related muscle dysfunction, we further examined its effects on fibre morphology. H&E staining (Figure 2A) showed that LIPUS markedly increased myofiber CSA relative to the aged group (Figure 2B). Fibre size distribution analysis demonstrated that the proportion of small fibres was substantially higher in the aged group, whereas the LIPUS group displayed a pronounced shift toward larger fibres (Figure 2C). To determine whether LIPUS influenced fibre type composition, immunofluorescence revealed an increase in myosin heavy chain (MyHC) type I (MyHC I) and a reduction in type IIa (MyHC IIa) after treatment (Figure 2D). These findings were corroborated by parallel changes in protein expression levels (Figure 2E), suggesting that LIPUS not only counteracts muscle atrophy but also promotes a fibre type shift that may enhance functional capacity. The protein levels and IHC staining of Atrogin‐1 and MuRF1 further verified the extent of muscle atrophy in this naturally ageing model. The results showed that the old group exhibited significantly higher expression of both Atrogin‐1 and MuRF1 compared to the young control group (Figure 2E). Notably, Atrogin‐1, but not MuRF1, expression was significantly reduced after LIPUS treatment relative to the old group. The IHC findings were consistent with the protein expression patterns (Figure 2F).

### 3.3 LIPUS Reduced AGEs Accumulation and Expression of Inflammation‐Related Markers in the Gastrocnemius of Naturally Aged Mice

**FIGURE 3:**
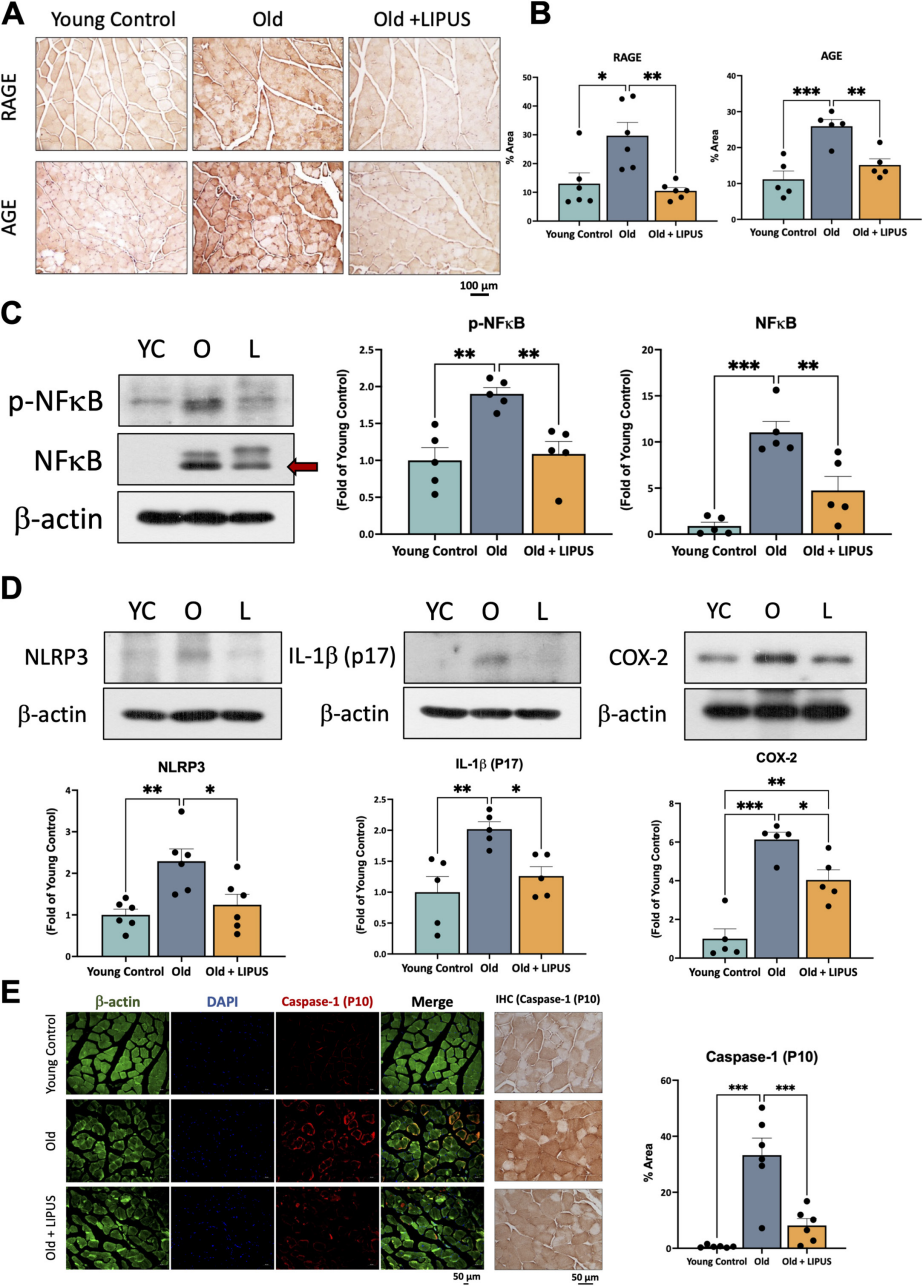
Increased accumulation of AGE and its receptor with enhanced inflammatory responses in the gastrocnemius of naturally aged mice. Gastrocnemius were collected after sacrifice, embedded in paraffin and sectioned for IHC staining. Protein distribution was analysed, and quantification was performed using ImageJ. (A) Representative IHC images of AGE and RAGE (20 × objective; scale bar = 100 μm). (B) Quantification of AGE and RAGE IHC staining. (C) Representative Western blot images and corresponding quantitative analysis of p‐NFκB and NFκB protein expression in the gastrocnemius. (D) Protein expression levels and quantification of inflammation‐related markers, including NLRP3, IL‐1β (P17), and COX‐2. (E) Caspase‐1 (P10) expression in Gastrocnemius, assessed by immunofluorescence (IF; 20 × objective, scale bar = 50 μm) and IHC (40 × objective, scale bar = 50 μm), along with quantification. Green represents β‐actin, blue represents DAPI, and red represents activated Caspase‐1. *, *p* < 0.05; **, *p* < 0.01; ***, *p* < 0.001.

Because serum markers of renal dysfunction were elevated in aged mice, we suspected reduced toxin clearance leading to tissue accumulation. Uremic toxins such as AGEs are well‐recognized indicators of kidney impairment. AGE accumulation is also known to be linked to ageing and age‐related diseases. We therefore performed IHC to assess AGE accumulation and expression of their receptor RAGE in gastrocnemius muscle (Figure 3A). Both representative images and quantitative analysis demonstrated a striking increase in AGE and RAGE expression in aged mice relative to young controls (Figure 3B). LIPUS treatment significantly decreased the expression levels of AGE and RAGE proteins. NF‐κB is one of the downstream signalling pathways activated by AGEs. The protein expression levels of both NF‐κB and p‐NF‐κB were significantly higher in the old group than in the young control group, whereas LIPUS treatment markedly reduced their expression (Figure 3C).

We next evaluated senescence‐associated proteins to characterize the muscle condition in this model. Quantitative analysis revealed significant upregulation of p21 and p53 in the gastrocnemius muscle of the aged group compared with young controls. LIPUS reduced the protein levels of p53 and p21 expression (for p53, *p* < 0.05; for p21, *p* = 0.139; Figure S2). These results confirm that the model exhibits ageing features and that LIPUS can attenuate them.

To investigate downstream pathways associated with AGE accumulation, we examined inflammation‐related markers. Compared with young controls, aged mice showed significantly elevated NLRP3 and activated IL‐1β and COX‐2. Notably, LIPUS markedly lowered the expression of these inflammatory proteins (Figure 3D). In addition, IHC and IF were used to evaluate activated caspase‐1, a downstream component of the NLRP3 inflammasome (Figure 3E). Expression of activated caspase‐1 was significantly increased in the aged group but was effectively reduced by LIPUS.

### 3.4 LIPUS Reduced the Systemic Inflammation Markers Expression and Modulated Apoptosis‐Related Proteins in Aged Skeletal Muscle

**FIGURE 4:**
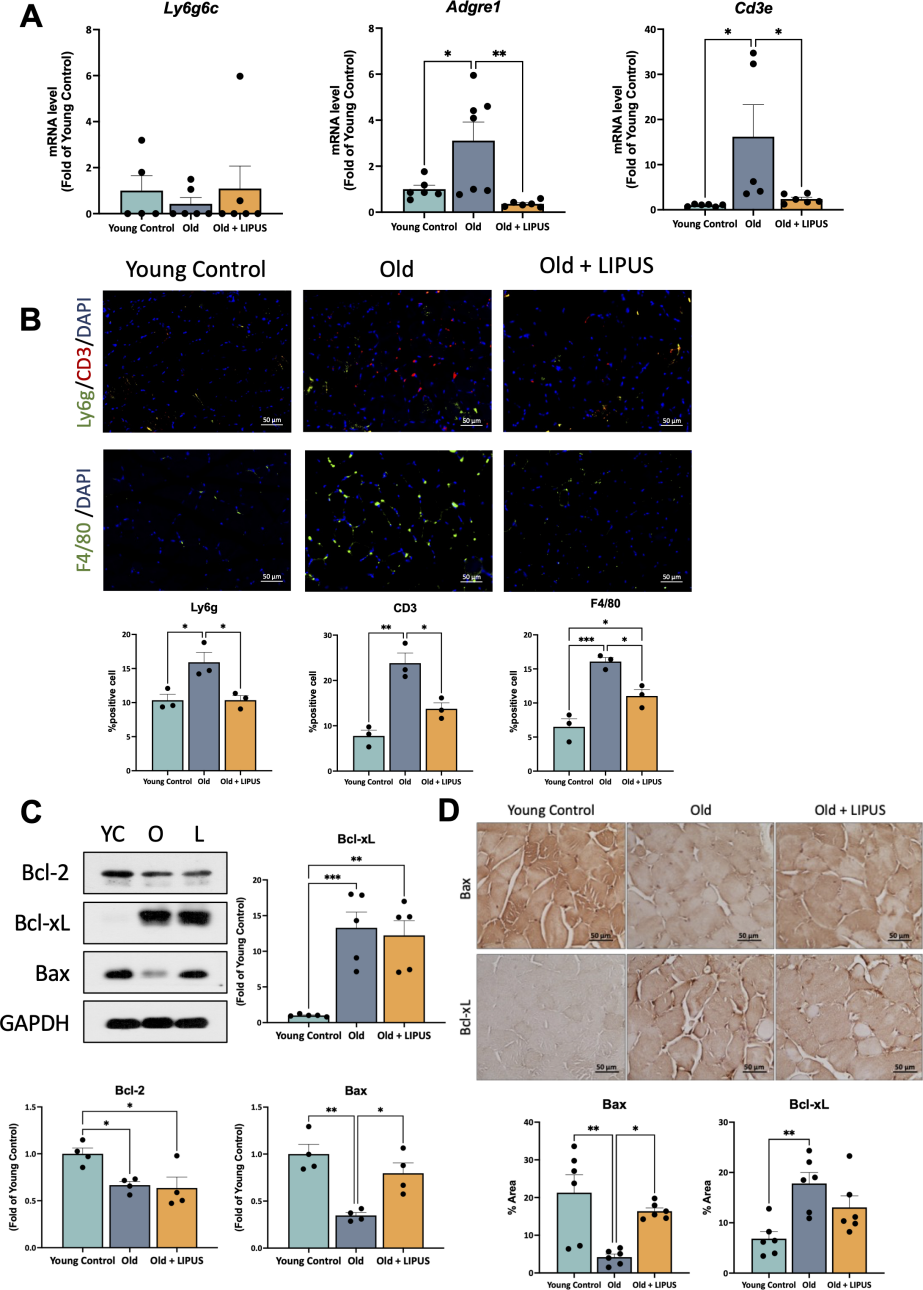
LIPUS reduced the expression of the systemic inflammation markers and modulated apoptosis markers in the gastrocnemius. 92‐week‐old mice received LIPUS treatment for 8 weeks. The gastrocnemius muscle after sacrifice was divided into two portions: one part was fixed and paraffin‐embedded for subsequent multiplex immunohistochemistry (mIHC) analysis, while the other part was snap‐frozen and stored at −80°C for subsequent mRNA and protein expression analyses, and quantification was performed using ImageJ. (A) The mRNA expression levels of systemic inflammatory markers, including Ly6g6c, Adgre1 and Cd3e, in the Gastrocnemius. (B) Representative images of tissue sections scanned using the TissueGnostics TissueFAXS system and analysed with the StrataQuest Analysis System for flow cytometry–like quantification of F4/80, Ly6g and CD3 expression across whole tissue sections. (C) Protein expression levels and quantification of inflammation‐related markers, including Bcl‐2, Bcl‐xL and Bax, in Gastrocnemius. (D) IHC for Bcl‐xL and Bax (40 × objective, scale bar = 50 μm), along with quantification. *, *p* < 0.05; **, *p* < 0.01; ***, *p* < 0.001.

To support the finding that LIPUS alleviates inflammatory indicators, we further analysed systemic inflammation markers, including Ly6g, F4/80 and CD3. The mRNA expression results revealed that Adgre1 and Cd3e were significantly upregulated in the old group compared to the young control group and were significantly downregulated following LIPUS treatment (Figure 4A). In contrast, Ly6g6c showed no significant differences across all groups (Figure 4A). The whole tissue sections scanned by using the TissueGnostics TissueFAXS system (Figure S3) were utilized to detect the expression of these systemic inflammation markers and analysed with the StrataQuest Analysis System for flow cytometry—like quantification (Figure 4B), yielding results consistent with the mRNA data. These findings support a potential for LIPUS in mitigating AGE‐associated inflammation.

The protein expression of anti‐apoptotic markers Bcl‐2 and Bcl‐xL showed opposite trends in the old group compared with the young control group, with both differences reaching statistical significance (Figure 4C). In contrast, the pro‐apoptotic protein Bax was significantly lower in the old group than in the young controls (Figure 4C,D). Following LIPUS treatment, a significant increase was observed only in Bax expression compared with the old group (Figure 4C,D). These findings suggest that apoptosis may not yet be activated in the muscle tissue of aged mice and that the aged muscle may have initiated compensatory responses to maintain fibre survival under the stress of ageing.

To further investigate whether the downstream apoptotic pathways were activated, protein expression was examined. Total caspase‐3 and caspase‐7 levels were significantly decreased in the old group compared to the young control group, whereas the cleaved forms of both caspases showed no differences (Figure S4). Following LIPUS treatment, total caspase‐7 expression was significantly higher than in the old group, but it did not significantly affect the cleaved forms of both caspase‐3 and caspase‐7 (Figure S4). These results indicated that classical caspase‐dependent apoptosis was not evidently activated in the aged muscle.

### 3.5 Natural Ageing Altered Gut Microbial Composition in Mice

In this natural ageing model, we observed classic features of muscle ageing and activation of inflammation‐related pathways, which may be driven by AGE accumulation. Strikingly, these age‐associated changes were significantly blunted by LIPUS treatment. Because LIPUS was applied to the abdomen, we further explored whether its beneficial effects involved the gut microbiota.

**FIGURE 5:**
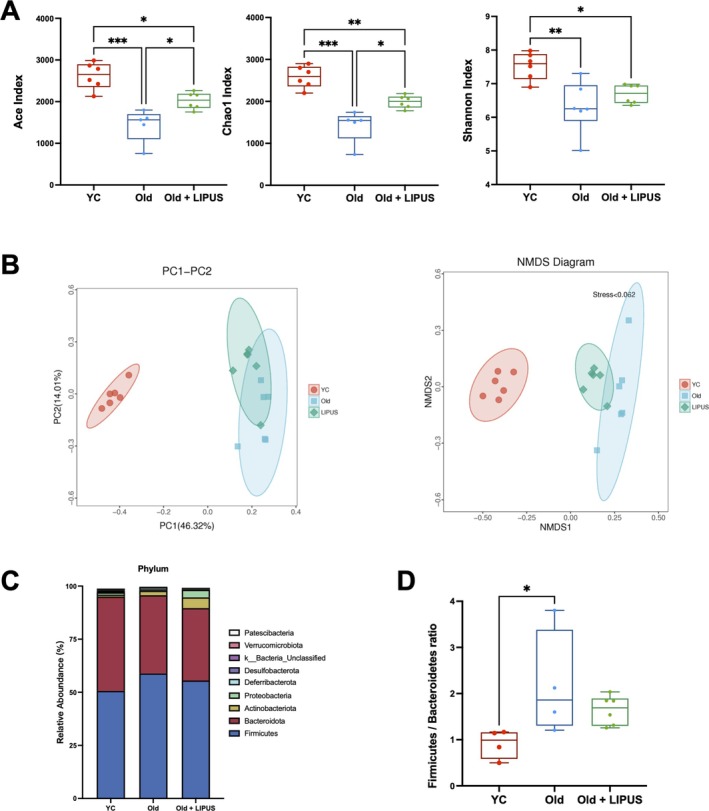
LIPUS increased gut microbiota richness and evenness and altered microbial composition in aged mice. Faecal samples were collected from the distal gut after sacrifice and outsourced for genomic DNA extraction and subsequent analysis. (A) Alpha‐diversity indices, including Ace, Chao1 and Shannon. (B) Beta‐diversity assessed by Principal Coordinate Analysis (PCoA) and nonmetric multidimensional scaling (NMDS). (C) Phylum‐level composition of gut microbiota (D) Firmicutes/Bacteroidetes (F/B) ratio. *, *p* < 0.05; **, *p* < 0.01; ***, *p* < 0.001.

Alpha‐diversity indices (Figure 5A), including Ace, Chao1 and Shannon, showed that ageing reduced microbial richness, whereas LIPUS restored diversity metrics. Beta‐diversity analysis (PCoA and NMDS) revealed distinct microbial community compositions among groups (Figure 5B), with statistical significance confirmed by Adonis testing (Table S2; young vs. old, *p* = 0.005; old vs. old + LIPUS, *p* = 0.03). ANOSIM analysis (Figure S5, Table S3) yielded consistent results. These data indicate that ageing disrupts gut microbiota composition and lowers diversity, while LIPUS reverses these effects.

Taxonomic profiling at the phylum level identified Firmicutes, Bacteroidota and Actinobacteriota as the most abundant phyla (Figure 5C). We also examined the Firmicutes/Bacteroidetes (F/B) ratio, a common gut health indicator (Figure 5D). The F/B ratio was significantly increased in aged mice relative to young controls, suggesting an age‐related decline in gut microbial health.

### 3.6 LIPUS Altered the Gut Microbial Profile in Naturally Aged Mice

**FIGURE 6:**
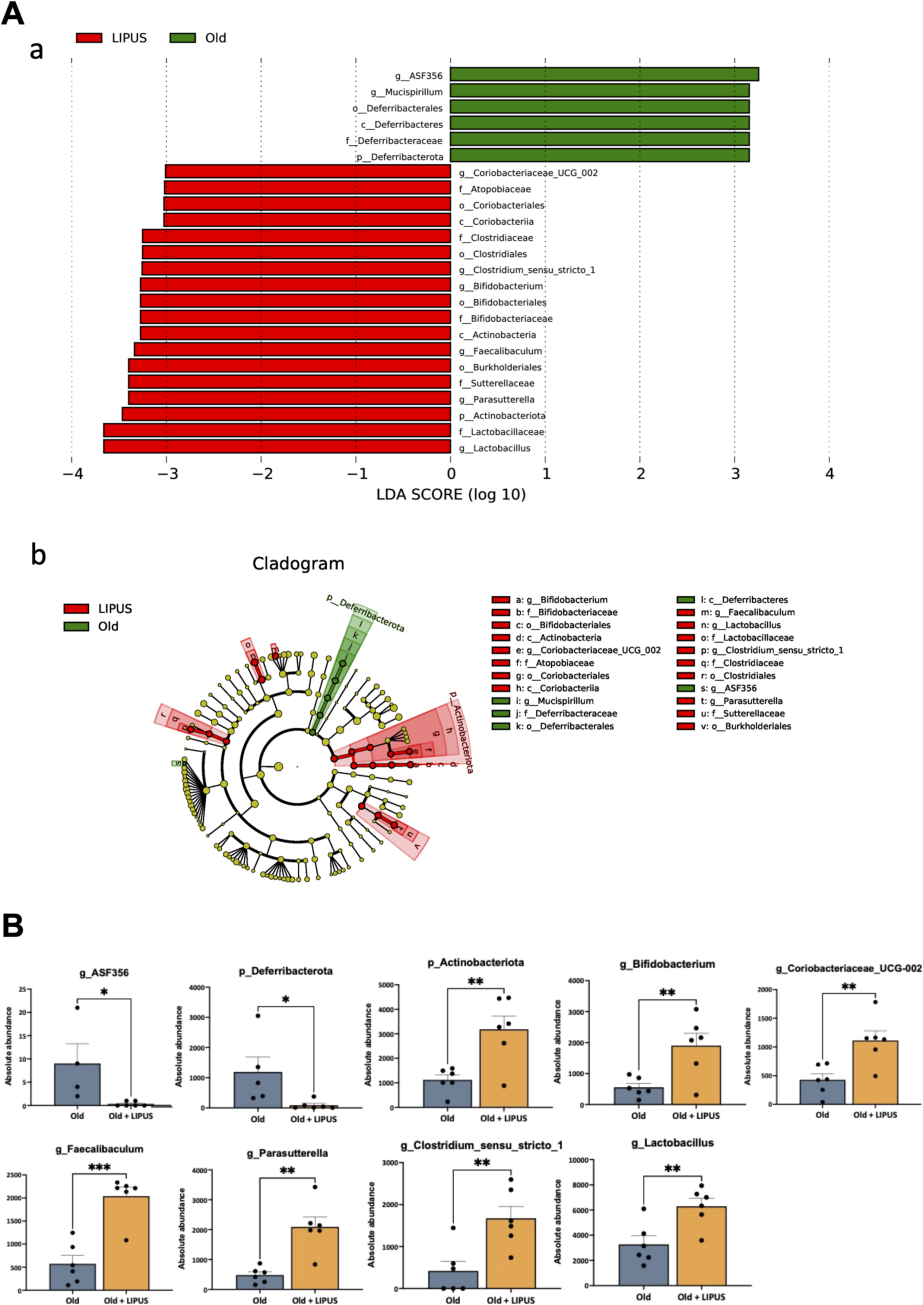
LIPUS increased beneficial gut microbiota in aged mice. Faecal samples were collected from the distal gut after sacrifice and outsourced for genomic DNA extraction and subsequent analysis. Linear discriminant analysis effect size (LEfSe) analysis was performed to identify differential taxa between old and LIPUS‐treated groups. (A) Bar plot showing LDA scores and a cladogram illustrating the hierarchical relationships of differential taxa. (B) Quantification of differential taxa between old and LIPUS‐treated groups. *, *p* < 0.05; **, *p* < 0.01; ***, *p* < 0.001.

To compare microbial differences between the aged and LIPUS groups, we performed linear discriminant analysis effect size (LEfSe) analysis (Figure 6A). The aged group was enriched with taxa such as g_ASF356 and other members of p_Deferribacterota, with which these microbes were often associated with chronic inflammation [11, 12] (Figure 6B). In contrast, the LIPUS group was enriched in beneficial genera including g_Bifidobacterium, g_Coriobacteriaceae_UCG_002, g_Faecalibaculum, g_Parasutterella, g_Clostridium_sensu_stricto_1 and g_Lactobacillus (Figure 6B). These taxa are recognized probiotics with anti‐inflammatory properties and the ability to enhance gut barrier integrity and modulate immune responses [13, 14, 15]. These observations suggest that LIPUS may ameliorate age‐related dysbiosis and inflammation by fostering the growth of beneficial bacteria.

**FIGURE 7:**
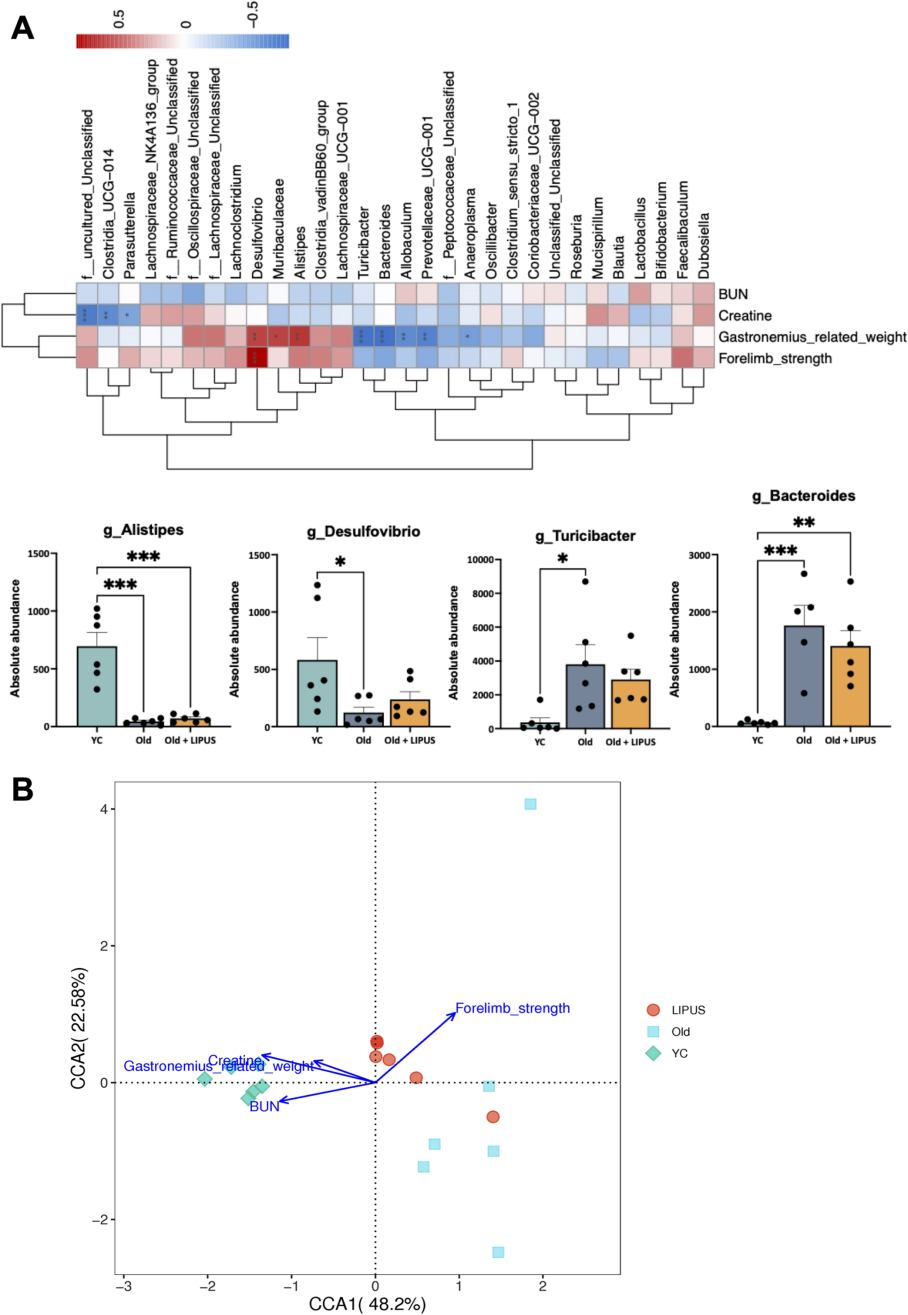
LIPUS increased beneficial gut microbiota in aged mice and showed positive correlations with muscle function. Faecal samples were collected from the distal gut after sacrifice and outsourced for genomic DNA extraction and subsequent analysis. Linear discriminant analysis effect size (LEfSe) analysis was performed to identify differential taxa between old and LIPUS‐treated groups. (A) Correlation analysis between differential taxa and physiological variables, including BUN, creatinine, relative weight of Gastrocnemius, and forelimb strength. (B) CCA plot showing the relationships between physiological variables (BUN, creatinine, relative weight of Gastrocnemius, forelimb strength) and sample groups. *, *p* < 0.05; **, *p* < 0.01; ***, *p* < 0.001.

We also compared microbiota between the young and aged groups (Figure S6), which showed significant increases in g_Turicibacter and g_Allobaculum in aged mice. Correlation analyses between physiological variables and gut microbiota revealed that Alistipes and Desulfovibrio were more abundant in young controls than in aged mice and positively correlated with muscle mass and forelimb strength (Figure 7A). By contrast, *Turicibacter* and *Bacteroides* were enriched in aged mice and negatively correlated with muscle mass (Figure 7A).

Canonical correspondence analysis (CCA) was conducted to explore the relationship between microbial composition and physiological parameters across groups (Figure 7B). The CCA plot showed clear separation of microbial communities, with the LIPUS group clustering separately from both young and old groups, indicating a major shift in community structure after treatment. Blue arrows representing physiological parameters pointed toward the LIPUS cluster, suggesting that improved muscle strength was strongly associated with the altered microbiota. Collectively, these findings indicate that LIPUS significantly modulates gut microbial composition, fostering a community more closely linked to enhanced muscle function.

## 4 Discussion

This study utilized a natural ageing mouse model to thoroughly examine age‐associated muscle deterioration and implemented LIPUS to the abdominal region to explore how the gut–muscle axis influences muscle function during ageing. In this model, we confirmed characteristic ageing phenotypes and detected the accumulation of AGEs in skeletal muscle, which may stem from renal impairment. Moreover, the gut microbiota showed pronounced shifts in older mice compared with young controls. Remarkably, LIPUS enhanced muscle performance, lessened ageing features and redirected gut microbial composition toward a profile considered advantageous for muscle health. Together, these outcomes highlight the therapeutic promise of LIPUS in mitigating sarcopenia by modulating the gut–muscle axis.

Chemically induced models, such as the D‐galactose ageing mouse [16], and genetically modified strains, including the SAMP8 [17], Rps9 D95N [18] and progeria‐like mice [19], are widely used to study sarcopenia‐related ageing phenotypes [20]. These systems facilitate mechanistic analyses and rapid intervention testing by accelerating ageing, shortening experimental timelines, reducing costs and generating distinct phenotypes. Genetic models additionally permit the dissection of gene‐specific mechanisms. However, because they artificially hasten the ageing process [21], they may not reproduce natural ageing pathology in its entirety. In contrast, natural ageing models offer a closer approximation to clinically relevant phenotypes such as sarcopenia and age‐related disorders [21]. Previous studies generally used mice 22–27 months of age [22, 23, 24], whereas in our study, ageing features were already apparent at 92 weeks of age (about 23‐month‐old), including hair loss and reduced mobility. By 100 weeks, grip‐strength testing revealed marked declines, indicating impaired muscle function. Subsequent analysis showed an elevated proportion of small myofibers and reduced muscle mass, typical of atrophy. Thus, the natural ageing model remains more physiologically representative and clinically meaningful.

As this study employed a naturally aged mouse model, neutrophil infiltration was not prominently elevated; therefore, Ly6g6c expression in mRNA level remained relatively low and did not differ significantly among groups. Previous research has indicated that while neutrophil infiltration remains low in the skeletal muscle of naturally aged mice under steady‐state conditions, it can be significantly exacerbated following acute injury compared to the young control [25]. In our study, we observed a distinct discordance between Ly6g mRNA and protein levels; specifically, Ly6g6c expression remained at basal or undetectable levels, while Ly6g protein was significantly upregulated in the old group. This discrepancy may reflect the dynamic nature of gene transcription during ageing. A transient upregulation of Ly6g6c at an earlier stage might have promoted subsequent translation and accumulation of Ly6g protein in skeletal muscle, even if mRNA levels were not consistently elevated at the time of measurement. Given that protein degradation machinery often became impaired in the old group, the persistent detection of Ly6g protein likely reflected its accumulation over time, explaining the inconsistency with current mRNA levels and highlighting the chronic inflammatory state of the natural ageing model, which LIPUS treatment effectively targets.

In the old group, we observed distinct alterations in apoptosis‐related proteins, including reduced Bcl‐2, elevated Bcl‐xL and decreased Bax. In combination with the lack of changes in cleaved caspase‐3 and caspase‐7, these findings suggest that the canonical apoptotic cascade is not engaged in this natural ageing model. A previous report indicated that Bcl‐xL expression can be transcriptionally regulated by NF‐κB, and elevated Bcl‐xL has been shown to suppress Bax activity [26]. This regulatory axis aligns with the reduced Bax levels detected in aged muscle. LIPUS selectively restored Bax expression toward levels observed in young muscle, whereas Bcl‐2 and Bcl‐xL were not affected, indicating a modest regulatory influence without initiating apoptotic activation.

Within the gut–muscle axis, short‐chain fatty acids (SCFAs) serve as key intermediaries linking gut microbes with muscle function [10]. Although SCFAs are absorbed by skeletal muscle and influence its mass [27], composition [28] and inflammatory state [29], our study did not directly quantify SCFA levels, which is a limitation. Nonetheless, LIPUS treatment increased SCFA‐producing taxa such as *Lactobacillus*, *Bifidobacterium* and *Clostridium* in aged mice, implying an anti‐inflammatory effect. SCFAs can suppress pro‐inflammatory cytokine release [30] and promote anti‐inflammatory T cell responses [11]. This mechanism aligns with our observations in aged skeletal muscle, where inflammatory markers (COX‐2, the NLRP3 inflammasome pathway and phosphorylated NF‐κB) were upregulated. Notably, LIPUS dampened these responses, likely through gut microbiota modulation that boosted SCFA production and reduced muscle inflammation.

Comparative analysis of gut microbiota between young controls and aged mice (Figures 7A and S6C) showed that *g_Turicibacter* and *g_Allobaculum* were significantly increased in aged animals. Both genera belong to Firmicutes and are associated with energy metabolism and SCFA production [31]. Specifically, g_Turicibacter has been reported to be highly correlated with anti‐inflammatory responses and the regulation of lipid metabolism [32], whereas *g_Allobaculum* mainly produces SCFAs, especially butyrate, supporting intestinal health [33]. Their pronounced increase in aged mice may reflect a compensatory attempt to maintain barrier integrity and energy balance, although over‐representation may also indicate age‐related microbial imbalance.

Current evidence regarding the impact of natural ageing on the gut microbiota remains incomplete, and our study contributes to this field by demonstrating marked alterations in microbial composition and diversity in aged mice compared with young controls. We identified specific microbial taxa altered in both aged mice and those receiving LIPUS treatment. These findings allow us to propose potential mechanistic links based on the known functions of these bacteria in naturally aged mice with or without LIPUS stimulation and suggest that future work could target these bacteria using selected probiotic strains to clarify the underlying mechanisms. A previous study has shown that long‐term supplementation with the multistrain probiotic ProBiotic‐4 (composed of *Bifidobacterium lactis* , *Lactobacillus casei* , *Bifidobacterium bifidum* , and *Lactobacillus acidophilus* ) improves gut microbiota–gut–brain axis dysfunction and cognitive deficits in aged senescence‐accelerated mouse prone‐8 (SAMP8) mice, likely by suppressing TLR4‐ and RIG‐I‐mediated NF‐κB signalling and inflammation [34]. Moreover, Chen et al. have also found that probiotic *L. casei* Shirota alleviated age‐associated sarcopenia in SAMP8 mice via the gut–muscle axis [35]. These previous studies could support the relevance of this direction. In the present study, although we did not test probiotic intervention directly or combination of LIPUS with probiotic, this remains a limitation of our study and an important direction for future research.

Prior studies have reported beneficial LIPUS effects on skeletal muscle. For example, under microgravity‐induced atrophy, LIPUS attenuated muscle loss [36]; in vivo, it accelerated regeneration via satellite cell proliferation, myotube growth and reduced fibrosis and inflammation. In vitro, LIPUS promoted mitochondrial biogenesis through AMPK activation and PGC‐1α induction, supporting satellite cell proliferation and differentiation [37]. LIPUS has also been shown to improve muscle recovery in postmenopausal women combined with PTH [38] and to suppress inflammation during acute injury by modulating WNT signalling and favouring M2 macrophage polarization [39]. Unlike most previous studies that applied LIPUS directly to muscle, our work targeted the abdominal region and demonstrated that LIPUS reshaped gut microbiota, expanded beneficial genera, improved muscle mass and function and tempered inflammatory signalling in aged mice. These findings suggest a new mechanism in which LIPUS acts through the gut–muscle axis, providing fresh insight into its therapeutic potential for sarcopenia.

**FIGURE 8:**
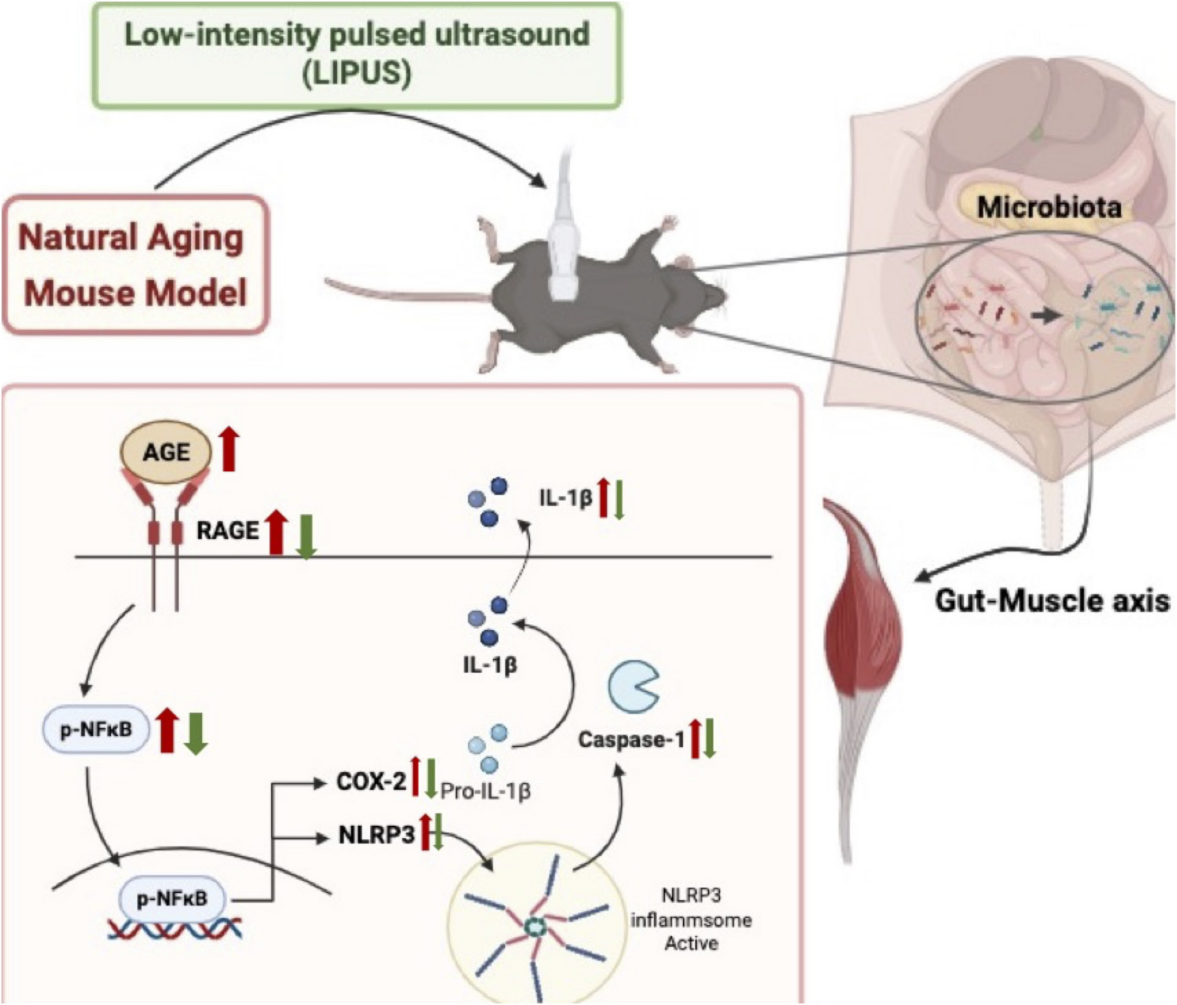
LIPUS alleviated AGE‐induced inflammatory responses in aged Gastrocnemius by modulating NFκB/NLRP3 inflammasome signalling and increasing beneficial gut microbiota. In 92‐week‐old mice, abdominal LIPUS treatment increased beneficial gut bacteria and enhanced microbial diversity. Furthermore, in the muscle, renal dysfunction caused by ageing led to the accumulation of AGE, which triggered inflammatory responses. LIPUS attenuated these effects by downregulating p‐NFκB and modulating the expression of NLRP3 inflammasome‐related proteins, thereby reducing caspase‐1 activation and IL‐1β release. In addition, LIPUS suppressed the expression of the pro‐inflammatory factor COX‐2, collectively alleviating muscle inflammation. AGE: advanced glycation end‐products; COX‐2: cyclooxygenase‐2; IL‐1β: interleukin‐1β; LIPUS: low‐intensity pulsed ultrasound; NFκB: nuclear factor kappa‐light‐chain‐enhancer of activated B cells; NLRP3: NOD‐, LRR‐ and pyrin domain‐containing protein 3; p‐NFκB: phosphorylated NFκB.

In conclusion, this study showed that the natural ageing mouse model induced sarcopenia‐like symptoms accompanied by inflammation and gut microbiota disruption, whereas abdominal LIPUS reversed or alleviated these alterations by suppressing inflammation and enriching beneficial bacteria (Figure 8). These results indicate that LIPUS is a safe, noninvasive approach with translational potential, meriting further investigation into microbiota–muscle interactions.

## Ethics Statement

The authors certify that they complied with the ethical guidelines for authorship and publishing of the Journal of Cachexia, Sarcopenia and Muscle (von Haehling S, Morley JE, Coats AJS, Anker SD. Ethical guidelines for publishing in the Journal of Cachexia, Sarcopenia and Muscle: update 2017. J Cachexia Sarcopenia Muscle 2017; 8: 1081–1083).

## Conflicts of Interest

The authors declare no conflicts of interest.

## Supporting information


**Figure S1:** The changes in body weight and kidney weight in aged mice.
**Figure S2:** LIPUS attenuated senescence markers in naturally aged mice.
**Figure S3:** Representative whole‐slide panoramic images acquired using the TissueGnostics TissueFAXS system and corresponding scatter plots for quantitative analysis.
**Figure S4:** Alterations in apoptotic markers in aged muscle.
**Figure S5:** Beta‐diversity analysis of gut microbiota reveals significant intergroup differences based on the ANOSIM test.
**Figure S6:** Differential gut microbiota between aged and young control groups.
**Table S1:** The primary antibodies used in the study.
**Table S2:** Beta‐diversity analysis of gut microbiota reveals significant intergroup differences based on the Adonis test.
**Table S3:** Beta‐diversity analysis of gut microbiota reveals significant intergroup differences based on the ANOSIM test.
**Table S4:** The primer sequences used in the study.
