# Supplementary material for: Gut Microbiota‐Linked Benefits of Low‐Intensity Pulsed Ultrasound Rejuvenate the Ageing Muscle
Source: J Cachexia Sarcopenia Muscle. 2026 Apr 21;17(3):e70291. doi: 10.1002/jcsm.70291 (PMC13096794; doi:10.1002/jcsm.70291)
Supplement: Supplementary file 1 — Figure S1: The changes in body weight and kidney weight in aged mice. Figure S2: LIPUS attenuated senescence markers in naturally aged mice. Figure S3: Representative whole‐slide panoramic images acquired using the TissueGnostics TissueFAXS system and corresponding scatter plots for quantitative analysis. Figure S4: Alterations in apoptotic markers in aged muscle. Figure S5: Beta‐diversity analysis of gut microbiota reveals significant intergroup differences based on the ANOSIM test. Figure S6: Differential gut microbiota between aged and young control groups. Table S1: The primary antibodies used in the study. Table S2: Beta‐diversity analysis of gut microbiota reveals significant intergroup differences based on the Adonis test. Table S3: Beta‐diversity analysis of gut microbiota reveals significant intergroup differences based on the ANOSIM test. Table S4: The primer sequences used in the study. [file JCSM-17-e70291-s001.docx]

**Supplementary Information**

**Gut Microbiota-Linked Benefits of Low-Intensity Pulsed Ultrasound Rejuvenates the Aging Muscle**

Jia-Hua Jhuang, Kuo-Cheng Lan, Ting-Yu Chang, Ding-Cheng Chan, Shing-Hwa Liu

**Figure S1. The changes in body weight and kidney weight in aged mice.**

C57BL/6 mice at ninety-two weeks of age were treated with LIPUS for 8 weeks before sacrifice. Body weight (A) and kidney weight (a)/relative kidney weight (b) (B) were shown. Data are presented as mean ± SD. *, p < 0.05; **, p<0.01; ***, p < 0.001.

**
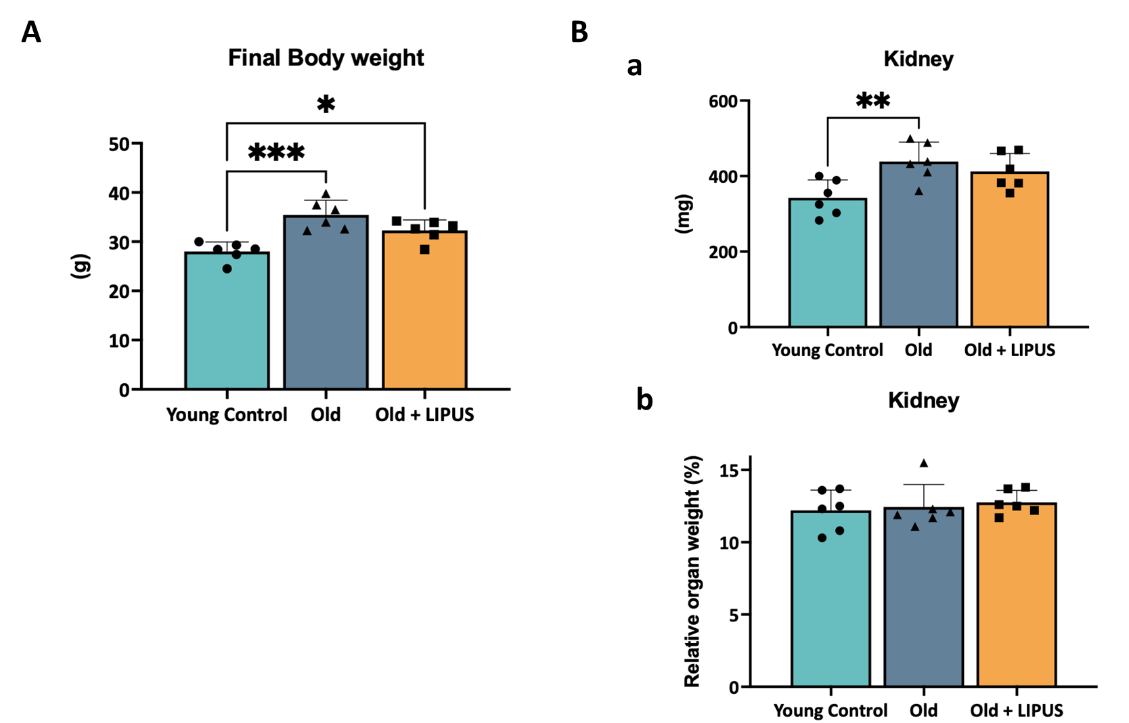
**

**Figure S2.** **LIPUS attenuated senescence markers in naturally aged mice.**

Ninety-two-week-old mice received LIPUS treatment for 8 weeks. Gastrocnemius were harvested at sacrifice and stored at –80 °C for subsequent protein analysis. Protein levels of P21 and P53 were analyzed by Western blot and quantified using ImageJ. Data are presented as mean ± SD. *, p < 0.05; **, p<0.01; ***, p < 0.001.


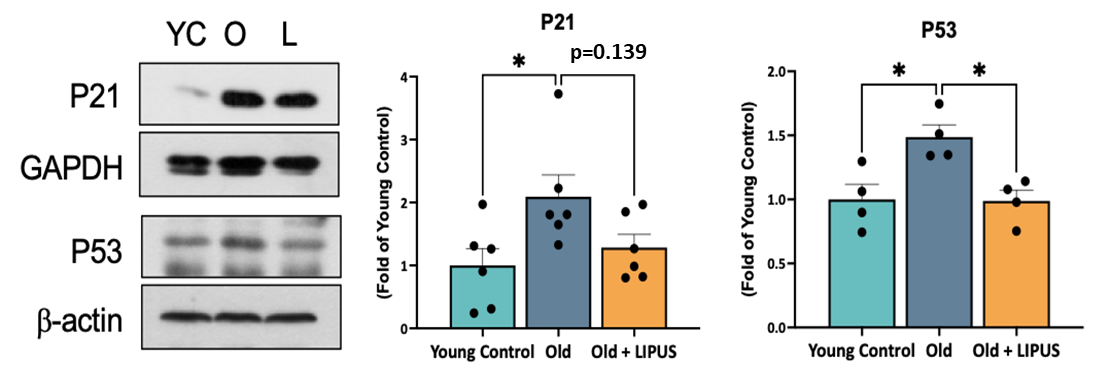


**Figure S3. Representative whole-slide panoramic images acquired using the TissueGnostics TissueFAXS system and corresponding scatter plots for quantitative analysis.**

The whole tissue sections scanned by using the TissueGnostics TissueFAXS system were utilized to detect the expression of these systemic inflammation markers and analyzed with the StrataQuest Analysis System for flow cytometry–like quantification. (A) Representative panoramic whole-section images illustrating Ly6g (green) and CD3 (red) expression. (B) Representative panoramic whole-section images illustrating F4/80 (green) expression. (C) Representative scatter plots of Ly6g (Marker1) and CD3 (Marker2) for quantitative analysis. (D) Representative scatter plots of F4/80 (Marker1) for quantitative analysis

(A)

**
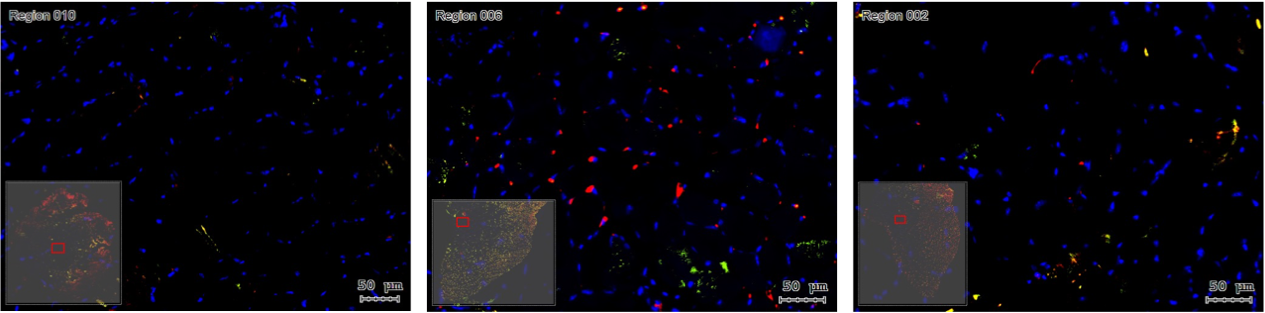
**

**(B)
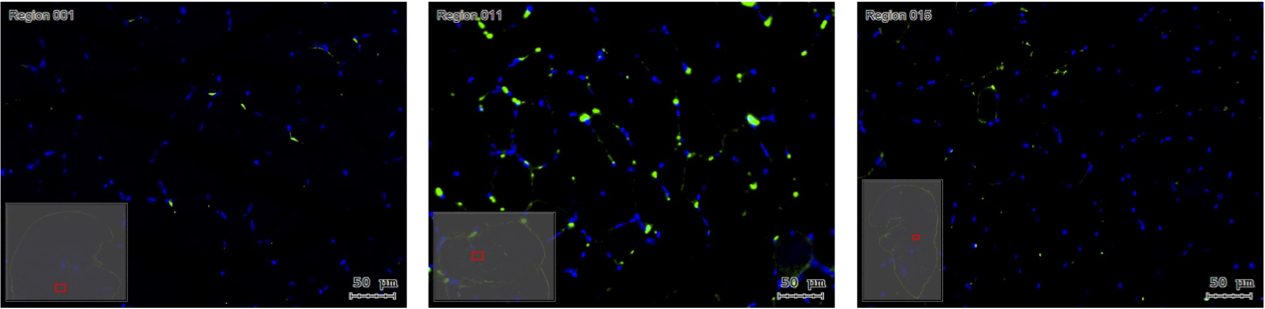
**

**(C)
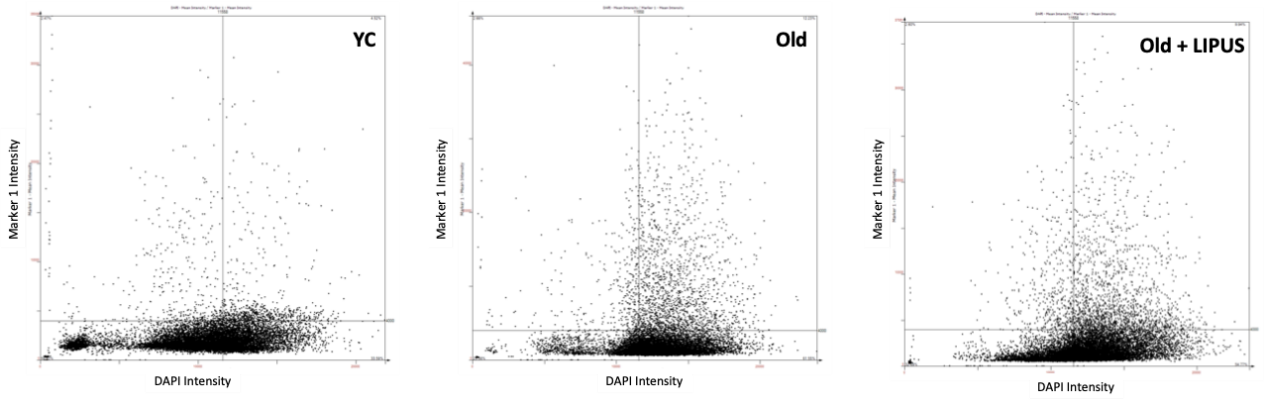
**

**(D)**


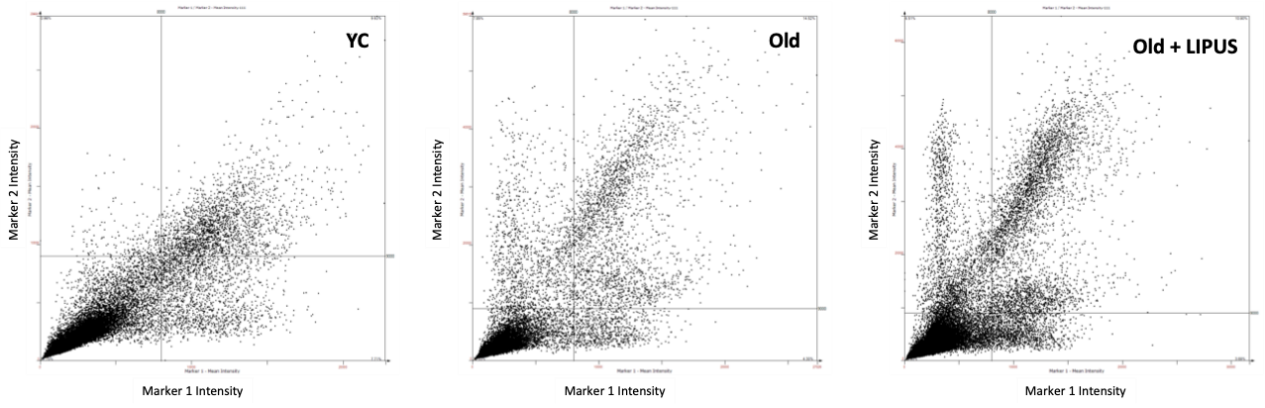


**Figure S4. Alterations in Apoptotic Markers in Aged Muscle**

Ninety-two-week-old mice received LIPUS treatment for 8 weeks. Gastrocnemius were harvested at sacrifice and stored at –80 °C for subsequent protein analysis. Protein levels of Caspase-3 and Caspase-7 were analyzed by Western blot and quantified using ImageJ. Data are presented as mean ± SD. *, p < 0.05; **, p<0.01; ***, p < 0.001.


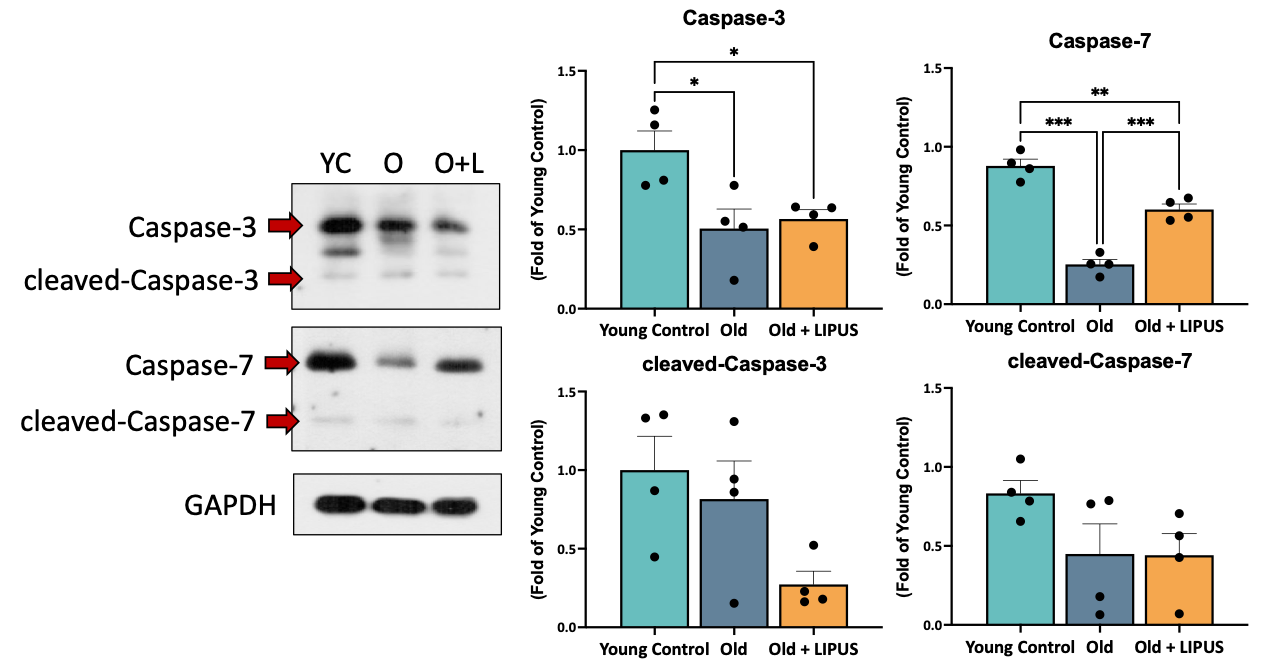


**Figure S5.** **Beta-diversity analysis of gut microbiota reveals significant intergroup differences based on the ANOSIM test.**

ANOSIM analysis showed significant intergroup differences (p < 0.05), with higher R values (ranging from 0 to 1) indicating greater separation between groups, as shown in the figure.


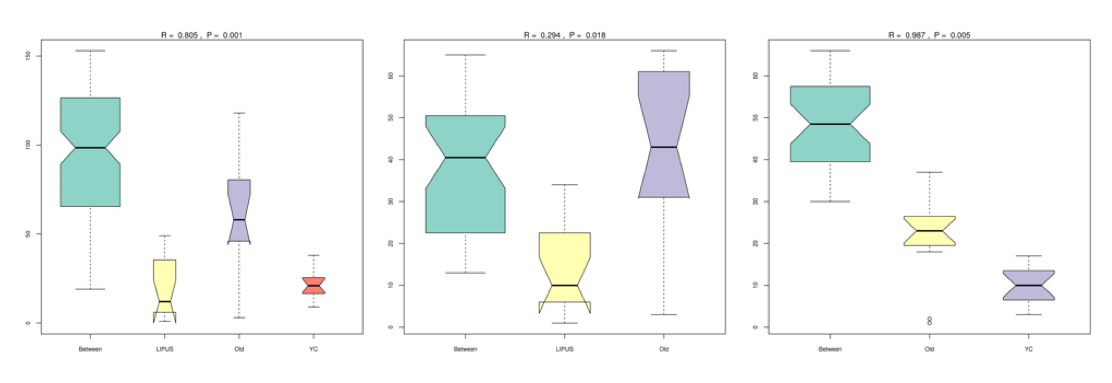


**Figure S6.** **Differential gut microbiota between aged and young control groups.** Fecal samples were collected from the distal gut after sacrifice and outsourced for genomic DNA extraction and subsequent analysis. LEfSe analysis was performed to identify differential taxa between the young control (YC) and the old groups.

A


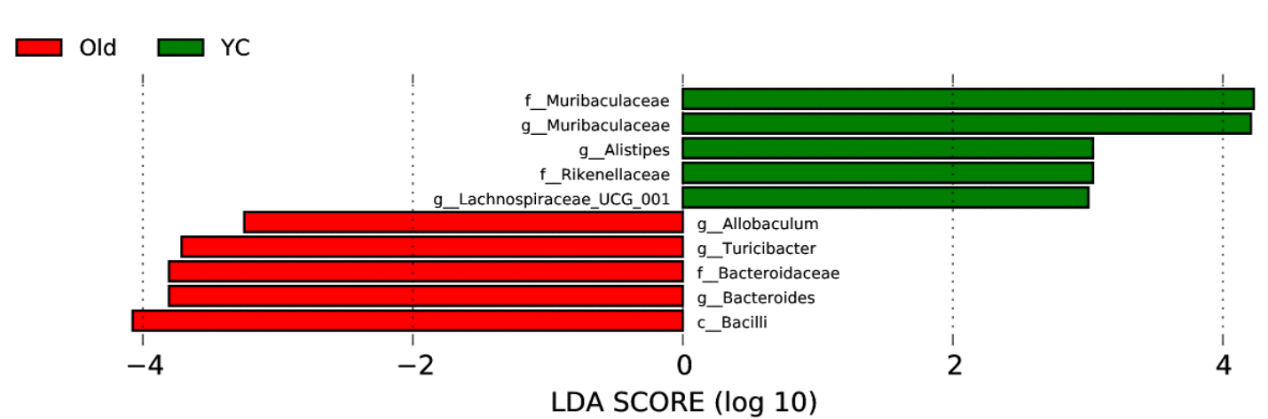


B


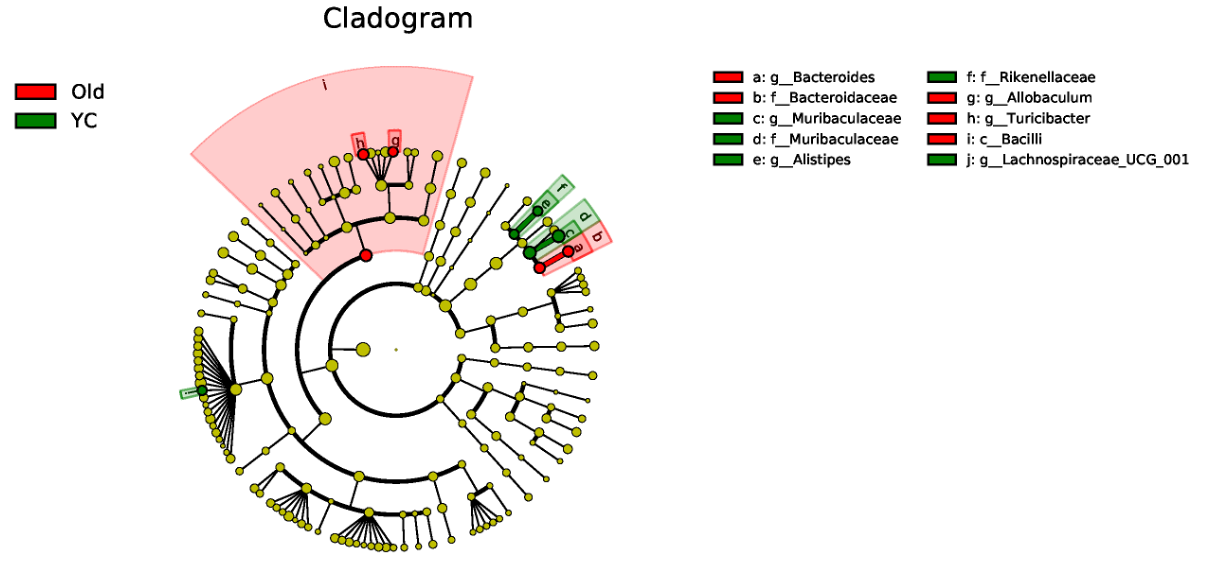


C


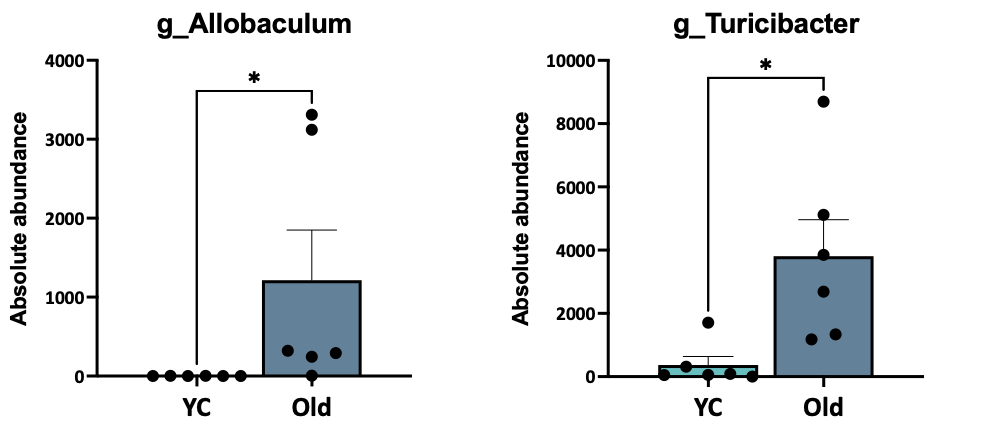


**Table S1. The primary antibodies used in the study.**

| Item | Brand | Serial number | Host species | Applications |
| --- | --- | --- | --- | --- |
| MyHC I | Millipore | M8421 | Mouse | IF, WB |
| Lamin B1 | Millipore | L9393 | Rabbit | IF |
| RAGE | Millipore | MAB5328 | Mouse | IHC |
| MyHC IIa | Santa Cruz | sc-53095 | Mouse | IF, WB |
| NFκB | Santa Cruz | sc-8008 | Rabbit | WB |
| IL-1β | Santa Cruz | sc-7884 | Rabbit | WB |
| β-actin | Santa Cruz | sc-47778 | Mouse | WB |
| MuRF1 | Santa Cruz | sc-398608 | Mouse | WB, IHC |
| Bax | Santa Cruz | sc-493 | Rabbit | WB, IHC |
| Bcl-xL | Santa Cruz | sc-8492 | Mouse | WB, IHC |
| P21 | Abcam | ab109199 | Rabbit | WB |
| NLRP3 | Abcam | ab263899 | Rabbit | WB |
| AGE | Abcam | ab23722 | Rabbit | IHC |
| Atrogin-1 | Abcam | ab157596 | Rabbit | WB, IHC |
| CD3 | Abcam | ab16669 | Rabbit | WB, mIHC |
| P53 | Cell Signaling | #32532 | Rabbit | WB |
| p-NFκB | Cell Signaling | #3033 | Rabbit | WB |
| Bcl-2 | Cell Signaling | #2870 | Rabbit | WB |
| GAPDH | Cell Signaling | #5174 | Rabbit | WB |
| COX-2 | BD | #610204 | Rabbit | WB |
| CF 568 | Biotium | - | - | IF |
| CF 488A | Biotium | - | - | IF |
| F4/80 | Invitrogen | 14-4801-82 | Mouse | WB, IHC, IF |
| Ly6g | MCE | HY-P86414 | Mouse | IF |

**Table S2.** **Beta-diversity analysis of gut microbiota reveals significant intergroup differences based on the Adonis test.**

After sacrifice, fecal samples were collected from the distal intestine for gut microbiota analysis. Beta-diversity was assessed to evaluate differences in microbial community structures among groups. Statistical analysis using Adonis revealed significant differences between groups (p < 0.05)

| **Groups** | **Df** | **R²** | **F-value** | **p-value** |
| --- | --- | --- | --- | --- |
| YC & Old & LIPUS | 2 | 0.543 | 8.913 | 0.005 ** |
| YC & Old | 1 | 0.519 | 10.776 | 0.005 ** |
| Old & LIPUS | 1 | 0.208 | 2.628 | 0.03 * |

**Table S3.** **Beta-diversity analysis of gut microbiota reveals significant intergroup differences based on the ANOSIM test.**

ANOSIM analysis showed significant intergroup differences (p < 0.05), with higher R values (ranging from 0 to 1) indicating greater separation between groups

| **Factor** | **R-value** | **p-value** |
| --- | --- | --- |
| YC & Old & LIPUS | 0.805 | 0.001 |
| YC & Old | 0.987 | 0.005 |
| Old & LIPUS | 0.294 | 0.018 |

**Table S4. The primer sequences used in the study**

| Gene Name | Sequences (5’- 3’) | |
| --- | --- | --- |
| Ly6g6c | Forward | TCTGCCCTACTCTACTGCTGG |
|  | Reverse | CCCACATCTTCCCAAGGTACA |
| Adreg1 | Forward | CGTGTTGTTGGTGGCACTGTGA |
|  | Reverse | CCACATCAGTGTTCCAGGAGAC |
| Cd3e | Forward | GAACCAGTGTAGAGTTGACGTG |
|  | Reverse | CCAGGTGCTTATCATGCTTCTG |
| Rpl13a | Forward | AGCCTACCAGAAAGTTTGCTTAC |
|  | Reverse | GCTTCTTCTTCCGATAGTGCATC |
